# A metagenomic insight into the microbiomes of geothermal springs in the Subantarctic Kerguelen Islands

**DOI:** 10.1038/s41598-022-26299-4

**Published:** 2022-12-23

**Authors:** Maxime Allioux, Stéven Yvenou, Alexander Merkel, Marc Cozannet, Johanne Aubé, Jolann Pommellec, Marc Le Romancer, Véronique Lavastre, Damien Guillaume, Karine Alain

**Affiliations:** 1 grid.466785.e Univ Brest, CNRS, IFREMER, IRP 1211 MicrobSea, Unité Biologie et Ecologie des Ecosystèmes Marins Profonds BEEP, IUEM, Rue Dumont d’Urville, 29280 Plouzané, France; 2 grid.465959.2 , Research Center of Biotechnology of the Russian Academy of Sciences, Winogradsky Institute of Microbiology, Moscow, Russia; 3 UBO, UFR Sciences et Techniques, UR 7462, Laboratoire Géoarchitecture, Territoires, Urbanisation, Biodiversité, Environnement, Rennes, France; 4 grid.4444.0 0000 0001 2112 9282 UJM, CNRS, LGL-TPE UMR5276, 42023 Saint Etienne, France

**Keywords:** Computational biology and bioinformatics, Ecology, Genetics, Microbiology

## Abstract

The Kerguelen Islands, located in the southern part of the Indian Ocean, are very isolated geographically. The microbial diversity and communities present on the island, especially associated to geothermal springs, have never been analyzed with high-throughput sequencing methods. In this article, we performed the first metagenomics analysis of microorganisms present in Kerguelen hot springs. From four hot springs, we assembled metagenomes and recovered 42 metagenome-assembled genomes, mostly associated with new putative taxa based on phylogenomic analyses and overall genome relatedness indices. The 42 MAGs were studied in detail and showed putative affiliations to 13 new genomic species and 6 new genera of *Bacteria* or *Archaea* according to GTDB. Functional potential of MAGs suggests the presence of thermophiles and hyperthermophiles, as well as heterotrophs and primary producers possibly involved in the sulfur cycle, notably in the oxidation of sulfur compounds. This paper focused on only four of the dozens of hot springs in the Kerguelen Islands and should be considered as a preliminary study of the microorganisms inhabiting the hot springs of these isolated islands. These results show that more efforts should be made towards characterization of Kerguelen Islands ecosystems, as they represent a reservoir of unknown microbial lineages.

## Introduction

Terrestrial hot springs are found all over the world, on all continents, and are abundant in areas of volcanic activity such as Iceland, Japan, Russia, Chile, Algeria or New Zealand^1,2^. With possible exception in the polyextreme Dallol area^3^, all studied geothermal environments harbor microbial cohorts. Microbial cohorts in terrestrial hot springs are often composed of *Bacteria* belonging to *Aquificae*,* Chloroflexi*,* Deinococcus*-*Thermus* and *Thermotogae*, and *Archaea* belonging to *Desulfurococcaceae*,* Thermoproteaceae* and *Thermococcaceae* (respectively referenced as follows in the Genome Taxonomy Database: *Aquificota*, *Chloroflexota*, *Deinococcota*, *Thermotogae*, *Desulfurococcaceae/Ignisphaeraceae*,* Thermoproteaceae*, and *Thermococcaceae*)^4–6^. Most of terrestrial hot springs, like those of Yellowstone (USA) or Kamchatka (Russia) areas, have been the subject of extensive microbiological investigations. These investigations include the study of the microbial community composition, the isolation and physiological characterization of microorganisms, the investigation of adaptive mechanisms of indigenous taxa, and the mining of extremophilic species for potential enzymes, activities or molecules of biotechnological interest (e.g.^7–12^). Studies of bacteria and archaea living in geothermal systems are essential for our knowledge of the history of life, as these environments are early Earth analogs and one of the possible cradles of life^2,6,13,14^. The microbial communities of hot springs in the polar regions are partly different in their composition. For example, the fumaroles of Deception Island (Antarctica) contain prokaryotic taxa belonging to *Verrucomicrobia*, *Proteobacteria*, *Planctomycetes*, ‘*Candidatus* Parcubacteria’, *Firmicutes*, *Chloroflexi*, *Calditrichaeota*, *Bacteroidetes*, *Thaumarchaeota*, *Nanoarchaeota*, *Euryarchaeota* and *Crenarchaeota* (respectively referenced as follows in the Genome Taxonomy Database: *Verrucomicrobiota*, *Proteobacteria*, *Planctomycetota*, *Patescibacteria*, *Firmicutes*, *Chloroflexota*, *Calditrichota*, *Bacteroidota*,* Thermoproteota*,* Nanoarchaeota*, *Methanobacteriota* and *Thermoproteota*)^15–17^. Due to their outstanding ecological and scientific values, specifically the presence of a wide diversity of rare endemic species or under-documented taxa, Deception Island and the other Antarctic geothermal ecosystems have been designated as Antarctic Special Protected Areas (ASPAs) by the Antarctic Treaty (management plan for Antarctic Specially protected area N° 175 and N° 140)^18^.

The volcanic Kerguelen Archipelago, which is part of the French Southern and Antarctic Lands, is situated in the southern part of the Indian Ocean (49° S, 69° E). Located at 3300 km from the nearest inhabited areas, it is amongst the most isolated islands from any continental landmass and contain a large part of the limited terrestrial habitats present at these latitudes. The Kerguelen Islands have the status of protected areas and are under the umbrella of international conventions supporting biodiversity protection (i.e., CITES, IUCN, Convention on Biological Diversity, IPBES). They represent an important UNESCO’s world heritage site and belongs to the national nature reserve (NNR) of the French Southern Lands. Such as the ASPAs, most of Kerguelen geothermal terrestrial micro-habitats are located within “strictly protected areas” (ZPI) set aside by the Nature Reserve, in order to protect biodiversity, and geological/geomorphological features. The Kerguelen Islands and the active volcanic Heard and MacDonald Islands are the only emerged entities among the vast Kerguelen oceanic Plateau. Kerguelen Archipelago is the third largest volcanic island complex in the world, after Iceland and Hawaii^19^. The last volcanic activity, dated 26 ± 3 Ka, took place on the Rallier du Baty (RB) Peninsula in the south-western part of the Kerguelen Islands^20^. Current volcanic activity, due to the Kerguelen hotspot, is evidenced by fumaroles, mud pots, hydrothermal discharges and small hot springs that rise from sea level to at least 300 m altitude. The waters of these geothermal systems contain large amounts of dissolved minerals and have wide pH ranges, from acidic to alkaline, and temperature ranges, from 35 to over 100 °C. The geochemical properties of the most accessible parts of this system have been monitored more or less regularly over the last decades^21,22^.

These geothermal habitats represent unique biodiversity sanctuaries in very insulated polar environments. Preliminary investigations based on 16S rRNA gene amplicon cloning and sequencing revealed a diverse collection of microbial community lineages composed of *Proteobacteria*, *Deinococcus-Thermus*, *Chloroflexi*, *Firmicutes*, *Actinobacteria* or *Aquificae*, as well as *Euryarchaeota*, *Crenarchaeota* (*Thermoproteales*,* Desulfurococcales*,* Acidilobales*,* Sulfolobales*) and *Thaumarchaeota* (respectively referenced as follows in the Genome Taxonomy Database: *Proteobacteria*, *Deinococcota*, *Chloroflexota*, *Firmicutes*, *Actinobacteriota*, *Aquificota*, *Methanobacteriota*, *Thermoproteota* and *Thermoproteota*)^23,24^. Some of these lineages were also found at geothermal sites in Antarctica, and others represented common taxa in geochemical environments worldwide. A small number of new species have also been isolated, enriched or highlighted through molecular approaches from these regions^23,25^. Apart from these few studies, this area has not been subjected to any comprehensive microbiological investigation to date. The microbial diversity hosted in these hot springs remains largely unknown, as well as its functional potential (metabolism, physiology, adaptations). Microbial communities might be shaped by the biogeographic position and the physicochemical parameters of the hot springs (temperature, pH, in situ chemistry), that probably exert a strong selective pressure on indigenous communities^17^. Yet, these geothermal springs represent undoubtedly unique diversity, and reservoirs of new functions and innovation.

**Figure 1:**
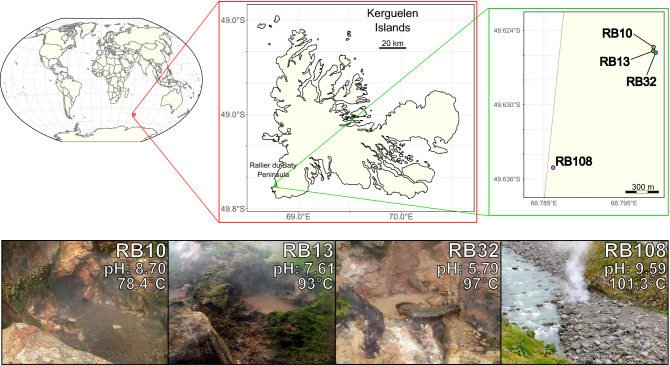
Sampling locations at the plateau des Fumerolles in the Rallier du Baty Peninsula (Kerguelen Islands, French Southern and Antarctic Lands), and photographs of the four hot springs studied here with their associated temperature and pH conditions. The world map and details of the Kerguelen seashore were generated using the ‘cowplot’, ‘ggplot2’, ‘lwgeom’, ‘rnaturalearth’, ‘rnaturalearthdata’, ‘rworldmap’, ‘sf’ and ‘tidyverse’ libraries implemented on the RStudio software (v1.2.5001). A 2018 map of the Kerguelen islands was retrieved from the French national portal of maritime limits (http://limitesmaritimes.gouv.fr/) according to the 2017 Etalab open license (v2.0—https://www.etalab.gouv.fr/). Pictures depicting sampling sites were taken by Marc Le Romancer. Layout was carried out using the free open source vector image editor Inkscape (v1.0).

In this study, we focused on four small geothermal hot springs from the Kerguelen Islands whose microbial communities have never been studied before. We analyzed the metagenomes of the hot springs referenced as RB10, RB13 and RB32, located on the "plateau des Fumerolles" at an altitude of about 300 m on the west coast of the Rallier du Baty Peninsula, and of the ephemeral spring referenced as RB108 which flows slightly above sea level into the riverbed of the Infernet glacier (located at the base of the plateau des Fumerolles) (Fig. 1). This work was carried out from samples collected with the aim of cultivating thermophilic microorganisms, which had been stored at 4 °C for 2 years. Those storage conditions induce biases and limit the scientific discussion. Therefore, the generated data did not allow ecological or evolutionary interpretations. These samples were collected in a particularly isolated natural reserve that requires specific sampling permits, and are therefore very difficult to obtain, making them particularly valuable. This descriptive study provides the first sequenced and analyzed MAGs from these undisturbed areas and points out that more efforts must be made to characterize Kerguelen Island ecosystems. This insular area contains a reservoir of unknown microbial lineages, and thus possibly biological and genomic novelties, the study of which may prove useful in the future to discover new metabolic pathways or molecules and to understand the part of deterministic and stochastic processes underlying microbial assemblages.

## Results and discussion

### MAG binning and general features

From the four hot springs, we assembled four associated metagenomes and then binned a total of 42 MAGs. We recovered 12 MAGs from RB10 hot spring, 13 from RB13, 14 from RB32 and 3 from RB108. Out of these 42 MAGs, 7 were of high-quality, 25 of nearly-high quality, 9 of medium quality and 1 of low quality (Table 1) based on metagenomic standards^26^. The GC% was quite variable, ranging from 25.76 to 70.35% among all MAGs and between 32.15 and 69.21% only among the high- and near high-quality MAGs. With the exception of RB108 from which we only recovered bacterial MAGs, we retrieved both bacterial and archaeal MAGs in the other hot springs. Two thirds of the MAGs (26/42) were assigned to the domain *Bacteria* and the rest to the domain *Archaea* (16/42) (Table 2).

**Table 1:**
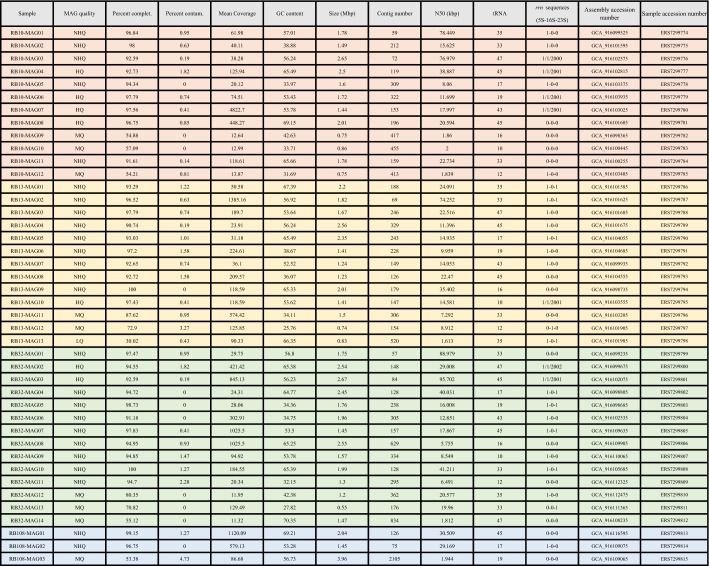
General characteristics of the 42 MAGs obtained from RB10, RB13, RB32 and RB108 samples.

In accordance with current standards, the bins were defined as high quality (HQ) (> 90% completion, < 5% contamination, presence of the 23S, 16S and 5S rRNA genes and at least 18 tRNAs), near high quality (NHQ) (> 90% completion, < 5% contamination, other criteria partially covered), medium quality (MQ) (≥ 50% completion, < 10% contamination), and low quality (LQ) (< 50% completion, < 10% contamination) MAGs. Metagenomes and MAGs accession numbers are available on ENA (Study ID: PRJEB46766).

**Table 2:**
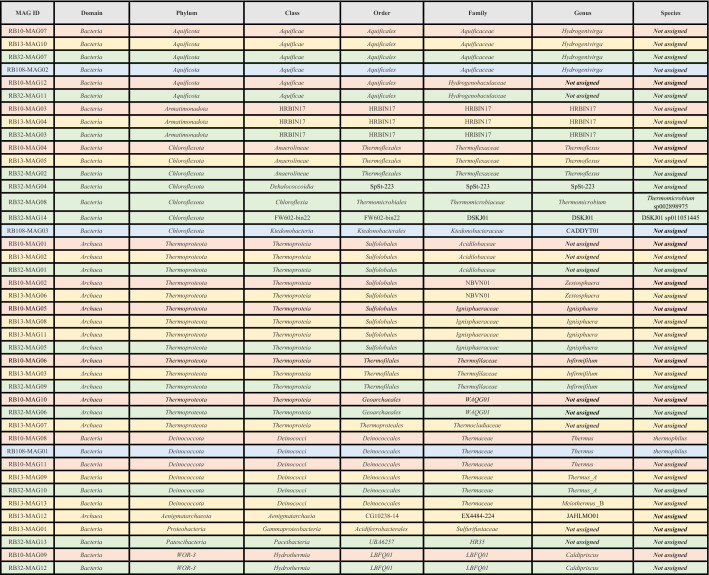
Classification of the MAGs based on the taxonomic classification of GTDB-Tk software (v2.1.0) and the Genome Taxonomy Database (07-RS207 release).

### Taxonomic and phylogenomic analyses of MAGs

The taxonomic affiliation of the MAGs was investigated in detail through the workflow classify of GTDB-Tk (v 2.1.0; GTDB reference tree 07-RS207) (Table 2) and through de novo phylogenomic analyses (Fig. S1a–i). We also tried to classify MAGs on the basis of overall genome relatedness indices (OGRI), which is detailed in supplementary material (Text S1, Table S2, Fig. S2).

De novo phylogenomic analyses globally confirmed the positioning of MAGs provided by GTDB-Tk, with high branching support. For *Bacteria*, GTDB-Tk analyses allowed us to place the MAGs in the following clades: six in the phylum *Aquificota* from the four different springs, comprising four MAGs belonging to the genus *Hydrogenivirga* (family *Aquificaceae*) (RB10-MAG07, RB13-MAG10, RB32-MAG07, RB108-MAG02), and two belonging to the family ‘*Hydrogenobaculaceae*’ (RB10-MAG12, RB32-MAG11) (Table 2, Fig. S1a). Their closest cultured relatives originated either from hot springs or from deep-sea hydrothermal vents^27^. Three MAGs from three geothermal springs belonged to the phylum *Armatimonadota* (RB10-MAG03, RB13-MAG04, RB32-MAG03) and had no close cultured relatives. Seven MAGs have been classified into the phylum *Chloroflexota:* three MAGs belonging to the genus *Thermoflexus* from three different springs (RB10-MAG04, RB13-MAG05, RB32-MAG02), one affiliating with the genus *Thermomicrobium* (RB32-MAG08), one falling into the family *Ktedonobacteraceae* (RB108-MAG03), one belonging to the class *Dehalococcoidia* (RB32-MAG04) and another one whose phylogenetic position is more difficult to assert because it is a MAG of medium quality (RB32-MAG14). Six MAGs from four various hot springs belonged to the phylum *Deinococcota*, and to the genera *Thermus* (RB10-MAG08, RB10-MAG11, RB13-MAG09, RB32-MAG10, RB108-MAG01) and *Meiothermus* (RB13-MAG13). One MAG belonged to the family ‘*Sulfurifustaceae*’ (RB13-MAG01), in the phylum *Proteobacteria* (*Gamma*-class). The MAG referenced as RB32-MAG13 was classified into the phylum ‘*Patescibacteria*’, in the class ‘*Paceibacteria*’, and was distantly related to MAGs originating from groundwater and from hot springs. Finally, two MAGs from two different springs belonged to the phylum WOR-3, in the *Candidatus* genus ‘Caldipriscus’ (RB32-MAG12, RB10-MAG09).

For *Archaea*, almost all the MAGs reconstructed in this study, e.g. 15 of the 16 archaeal MAGs, belonged to the phylum *Thermoproteota*. Among them, four belonged to the genus *Ignisphaera* (RB10-MAG05, RB13-MAG08, RB13-MAG11, RB32-MAG05), three to the genus *Infirmifilum* (RB10-MAG06, RB13-MAG03, RB32-MAG09), two to the genus *Zestosphaera* (RB10-MAG02, RB13-MAG06), three to the family *Acidilobaceae* (RB10-MAG01, RB13-MAG02, RB32-MAG01) and two to the order *Geoarchaeales* (RB10-MAG10, RB32-MAG06). Additionally, one belonged to the family *Thermocladiaceae* (RB13-MAG07). Lastly, the MAG belonging to another phylum (RB13-MAG12) was affiliated with the ‘*Aenigmatarchaeota*’, class ‘*Aenigmatarchaeia*’, and was distantly related to MAGs from hot springs and from deep-sea hydrothermal vent sediments^28,29^.

Out of these 42 MAGs, at least 19 MAGs corresponded to different taxa at the taxonomic rank of species or higher according to GTDB (Table 2). Eighteen of them belonged to lineages with several cultivated representatives including the species *Thermus thermophilus*. 13 new genomic species within the GTDB genera *Hydrogenivirga*, HRBIN17,* Thermoflexus*, SpSt-223, CADDYT01,* Zestosphaera*, *Ignisphaera*, *Infirmifilum*, *Thermus*, *Thermus_A*,* Meiothermus_B*, JAHLMO01 and *Caldipriscus*, and 6 putative new genomic genera belonging to the GTDB families *Hydrogenobaculaceae*,* Acidilobaceae*, WAQG01, *Thermocladiaceae*, *Sulfurifustaceae* and HR35 could be identified (Table 2). In addition, 9 MAGs belonged to lineages that are predominantly or exclusively known through environmental DNA sequences. Thus, these 42 MAGs comprised a broad phylogenetic range of *Bacteria* and *Archaea* at different levels of taxonomic organization, of which a large majority were not reported before.

**Figure 2:**
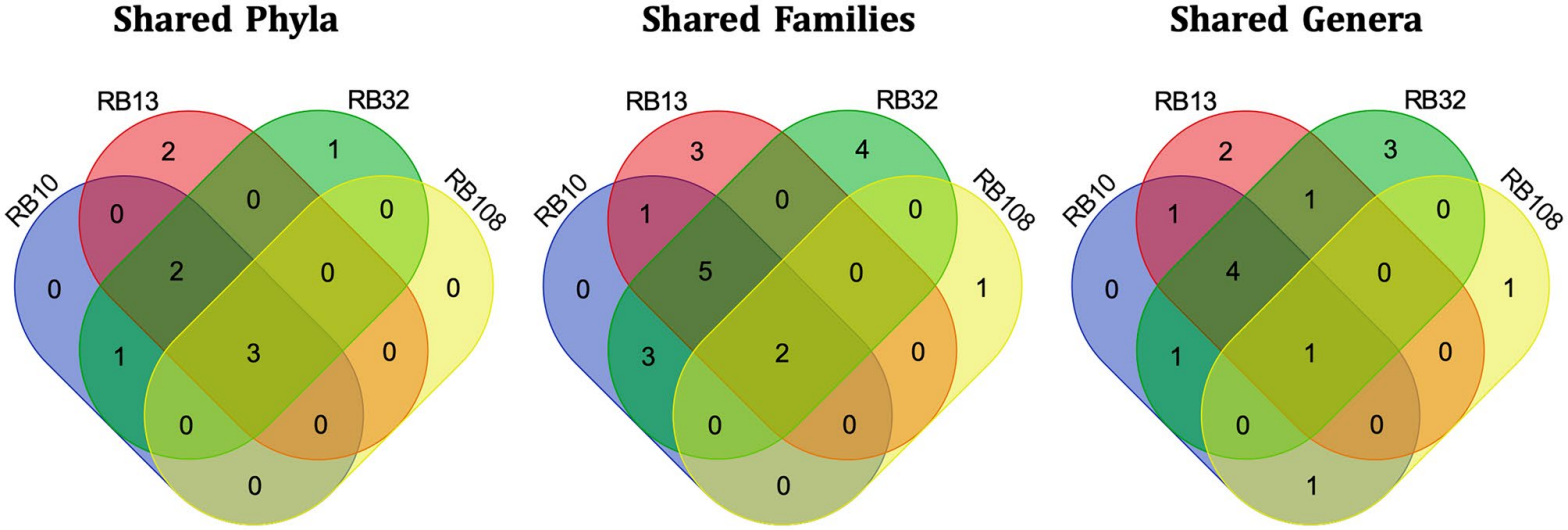
Venn diagrams showing the shared phyla, families and genera according to GTDB classification in the reconstructed MAGs from the hot springs RB10, RB13, RB32 and RB108.

The approaches implemented here were not intended to describe the microbial diversity present in these sources in an exhaustive way or to compare them in a fine way, and cannot allow it because of a 2-year storage at 4 °C. This long storage has probably led to changes in the microbial communities and to the selective loss or enrichment of some taxa. As a result, no analysis of abundance or absence of taxa can be conducted from these metagenomes and the results are discussed taking this bias into account. However, they do provide an overview of the microbial diversity effectively present. If we compare the phylogenetic diversity of the MAGs found in the four hot springs, we can observe that 3 shared phyla (*Deinococcota*, *Aquificota* and *Chloroflexota*: phyla names according to GTDB), 2 shared families (*Thermaceae* and *Aquificaceae*), and one shared genus (*Hydrogenivirga*) were found among the four sources (Fig. 2). In addition, hot springs RB10, RB13 and RB32, that are geographically close (< 60 m), also share 2 other phyla (*Thermoproteota* and *Armatimonadota*) and 5 other families in common (*Acidilobaceae*, *Ignisphaeraceae*, *Thermofilaceae*,* Thermoflexaceae*, and HRBIN17) (Fig. 2). These phyla and families that are shared between sources are widespread lineages in terrestrial geothermal habitats (e.g.^4–6,12^). Phyla and families detected in the hot environments of Antarctica are also found here, such as *Patescibacteria*^15^. In summary, this metagenomic analysis highlighted the presence of bacterial and archaeal lineages commonly found in hot springs, and lineages found in hot habitats from polar areas (e.g.^4–6,15,30^). The microbial communities in these Kerguelen Islands hot springs samples were diverse, particularly in RB10, RB13, and RB32 hot springs. Within these lineages previously reported to occur in geothermal environments, a majority of the genomic taxa detected here were novel. Those results were obtained considering their taxonomic affiliation by GTDB-Tk, and their phylogenomic position with respect to closest relatives and the OGRI thresholds (16S rRNA gene sequence similarity, average nucleotide identity, and average amino acid identity) classically used to delineate different taxonomic ranks in cultured strains, used here as indicators of taxonomic differentiation (Table 2, Table S2).

### Functional potential of MAGs: putative metabolisms and adaptations

**Figure 3:**
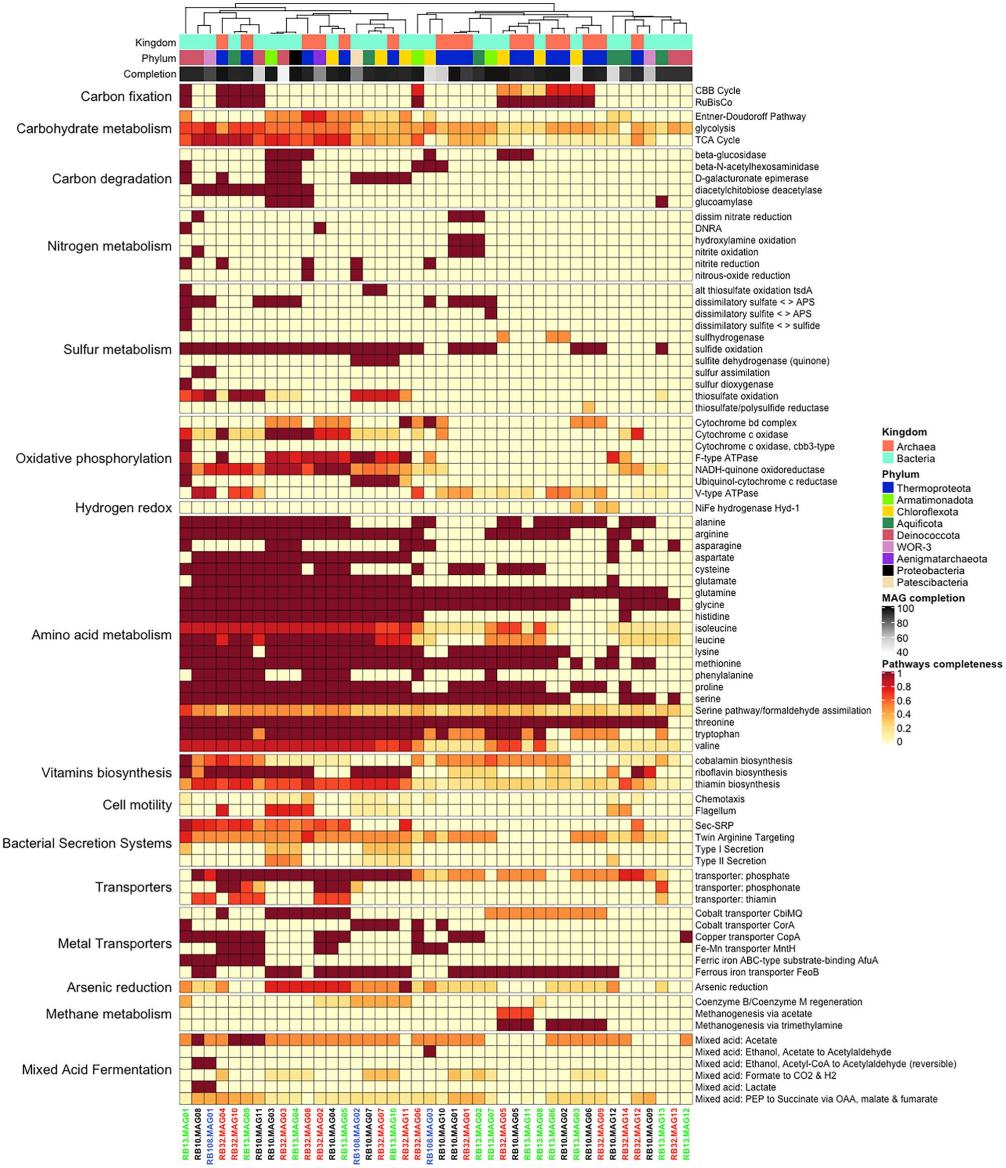
Metabolic pathway diagram of the 42 MAGs based on KEGG Decoder annotations, showing MAG classification according to GTDB-Tk and estimated genome completion.

A genomic characterization of the 42 MAGs has been performed to explore the possible metabolic pathways and adaptations of the microbial populations from which these MAGs originate. KEGG Decoder visualization highlighted various pathways associated with carbohydrate degradation, oxidative phosphorylation and sulfur, nitrogen, and amino-acid metabolisms, among others (Fig. 3). To confirm these initial metabolic predictions, a further annotation was performed by combining data generated by Prokka with the MetaCyc database. Efforts have been directed at studying catabolic pathways, particularly those involving inorganic electron donors and acceptors. These results are not representative of the metabolic diversity of all the hot spring ecosystems studied, but they do reflect some of the microbial catabolism likely to be used in situ to produce energy and, by assumption, the most abundant ones. Metabolic predictions are presented hereafter, at different taxonomic ranks and have been compared to the known phenotypes of the closest cultivated taxa, and in some cases to genomic content of the closest relatives.

MAGs belonging to the genus *Thermoflexus* (RB10-MAG04, RB13-MAG05, RB32-MAG02) encode pathways for carbon monoxide oxidation (via aerobic carbon monoxide dehydrogenase), hydrogen oxidation and nitrate respiration; the only cultivated known representative of this genus is a heterotrophic bacterium^31^. The same pathways, except for the nitrate reduction pathway, are encoded in the complete genome of *Thermoflexus hugenholtzii* (NCBI: ASM1877156v1). In contrast, the genome of *T*. *hugenholtzii*, a strain isolated from a terrestrial hot spring in Nevada^31^, encodes the tetrathionate reduction and thiosulfate disproportionation pathways, which are not encoded in the three Kerguelen Island MAGs. The *Dehalococcoidia*’s MAG (RB32-MAG04) encodes only a carbon monoxide oxidation pathway; the cultivated members of this genus are strict anaerobic hydrogenotrophic, organohalide-respiring bacteria^32^. In the MAG associated with the genus *Thermomicrobium* (RB32-MAG08), we predicted pathways for dimethylsulfide degradation, thiosulfate disproportionation and carbon monoxide oxidation; only carboxydotrophic growth has been reported in this genus and demonstrated by culture^33^. The same pathways are encoded in the complete genome of *Thermomicrobium roseum*, a strain isolated from a hot spring in Yellowstone National Park (NCBI: ASM2168v1)^34^. In the *Chloroflexota*’s MAG (RB32-MAG14) (belonging to the order *Chloroflexales*, Table S2), carbon monoxide oxidation and thiosulfate disproportionation pathways are present but no coding DNA sequence associated with phototrophy could be find, which may suggest a chemoorganotrophy mode of energy production^35^. The *Ktedonobacteraceae*’s MAG (RB108-MAG03) encodes enzymes for hydrogen oxidation (aerobic) pathways, carbon monoxide oxidation, dimethylsulfide degradation, selenate reduction, thiosulfate oxidation and disproportionation and finally tetrathionate oxidation; yet, the few taxa of this family isolated so far are mesophilic heterotrophic bacteria^36^. Within *Hydrogenobaculaceae* MAGs (RB10-MAG12, RB32-MAG11), we predicted a thiosulfate disproportionation pathway; most of the species within this family are capable of chemolithotrophic microaerophilic or anaerobic growth^37^. MAGs belonging to the genus *Hydrogenivirga* (RB10-MAG07, RB13-MAG10, RB32-MAG07, RB108-MAG02) possess genes encoding enzymes of aerobic respiration, thiosulfate oxidation, thiosulfate disproportionation, tetrathionate reduction, and hydrogen oxidation (aerobic and anaerobic); which is consistent with what is known about the genus (nitrate and oxygen respiration combined to hydrogen, sulfur, or thiosulfate oxidation)^37^. The same pathways, with the exception of the hydrogen oxidation pathway, are encoded in the genome of *Hydrogenivirga caldilitoris* (NCBI: ASM366400v1), a close relative isolated from a coastal hot spring in Japan^27^. In MAGs associated with the genus *Thermus* (RB10-MAG08, RB10-MAG11, RB13-MAG09, RB32-MAG10, RB108-MAG01), we predicted pathways for aerobic respiration, assimilatory sulfate reduction, hydrogen oxidation, selenate reduction, thiosulfate oxidation and thiosulfate disproportionation; cultivated species of this genus grow mainly chemoorganoheterotrophically by aerobic respiration, but some have genes coding for chemolithotrophic and anaerobic respiration enzymes^38^. The MAG belonging to the genus *Meiothermus* (RB13-MAG13) encodes pathways for carbon monoxide oxidation, hydrogen oxidation, thiosulfate oxidation and thiosulfate disproportionation; *Meiothermus* strains are known to grow chemoorganotrophically by oxygen or nitrate respiration^39^. For the RB13-MAG01 belonging to the *Sulfurifustaceae*, we predicted the genetic potential for aerobic respiration, ammonia oxidation, dissimilatory sulfate reduction, sulfite oxidation, sulfide oxidation (to sulfur globules), tetrathionate reduction, thiosulfate oxidation and thiosulfate disproportionation; *Sulfurifustaceae* (referenced as *Acidiferrobacteraceae* in the LPSN taxonomy) are known to be able to oxidize sulfur and iron, and the microorganism corresponding to this MAG may possess a larger panel of chemolithotrophic abilities^40^. For members of the *Armatimonadota* (RB10-MAG03, RB13-MAG04, RB32-MAG03), we predicted pathways for assimilatory sulfate reduction, carbon monoxide oxidation, selenate reduction and thiosulfate disproportionation; the members of the phylum have a phenotype of aerobic heterotrophs^41^. In *Zestosphaera*’s (RB10-MAG02, RB13-MAG06) and *Ignisphaera*’s (RB10-MAG05, RB13-MAG08, RB13-MAG11, RB32-MAG05) MAGs, we predicted sulfur and polysulfide reduction pathways; those MAGs could be classified as *Desulfurococcaceae* (LPSN taxonomy, Table S2) which are known as heterotrophs respiring sulfur species^42,43^. MAGs belonging to the class *Thermoproteia* (RB10-MAG10, RB13-MAG07, RB32-MAG06) encode dissimilatory sulfate reduction pathway; various catabolic pathways are described in this class^44^. In MAGs related to the genus *Caldipriscus* (RB10-MAG09, RB32-MAG12), phylum *Patescibacteria* (RB32-MAG13), family *Acidilobaceae* (RB10-MAG01, RB13-MAG02, RB32-MAG01), family *Thermofilaceae* (RB10-MAG06, RB13-MAG03, RB32-MAG09) and class *Aenigmatarchaeia* (RB13-MAG12), we did not predict any catabolic pathway of inorganic nutrients among those reported in the MetaCyc database. This could be explained by the low completion of the MAGs and/or the fact that only well-known pathways are documented in this database. However, all these MAGs have pathways associated with carbohydrate and protein degradation. This may indicate that these taxa are chemoheterotrophs, which has already been reported in geothermal environments and already described for relatives of some of these taxa^45,46^.

Sulfide oxidation may be a possible energy production pathway for 28 MAGs based on KEGG Decoder (Fig. 3), since they code for a sulfide:quinone oxidoreductase (KEGG:K17218) and a flavoprotein chain of sulfide dehydrogenase (KEGG:K17229), but this hypothesis was not confirmed by MetaCyc except for RB13-MAG01. Due to high representations of sulfur metabolisms, genes encoded in MAGs were evaluated with DiSCo, which gave similar results to those obtained when analyzed with Pathway tools. DiSCo confirmed complete dissimilatory sulfate reduction pathways for two MAGs, predicted to be associated to sulfate reduction processes (RB13-MAG07) or sulfide oxidation processes by reverse sulfate reduction pathway (RB13-MAG01). The assimilatory sulfate reduction pathway is more represented in the overall dataset formed by all MAGs than the dissimilatory pathway, which is consistent with the low sulfate concentration measured in the four hot springs (Table S1). The thiosulfate disproportionation pathway predicted by MetaCyc in many MAGs simply refers to the detection of an enzyme, the rhodanese-type thiosulfate sulfurtransferase. However, in the current state of knowledge on the disproportionation pathways of inorganic sulfur compounds^47,48^, this enzyme alone does not allow the implementation of this catabolic pathway. If we consider all the genes present in these MAGs, nothing indicates that the microorganisms from which these MAGs originate can achieve the disproportionation of inorganic sulfur compounds.

Additionally, no enzymes clearly associated with photosystems I and II were found. Nevertheless, it cannot be ruled out that these energy production pathways are absent in microorganisms indigenous to these sources, due to sample storage bias and low completion of some MAGs. On the other hand, our results show that these sources host chemolithoautotrophic taxa involved in the carbon and sulfur cycle, and to a lesser extent in the hydrogen and nitrogen cycles. Several taxa are likely to be involved in the primary production of these sources through chemolithoautotrophy, but in addition, heterotrophs appear to be present and diverse in the collected samples. Additional studies will be required to better understand the metabolic diversity and trophic webs of these hot springs, in order to better understand the ecology of the microbial communities of the Kerguelen hot springs.

Regarding thermophily, we found that all MAGs encode heat shock proteins, mainly associated with the HSP20 family, with the exception of RB10-MAG12 and RB13-MAG12. The absence of Hsp encoding genes in these two MAGs is possibly due to the low genome completeness of these two MAGs. Under conditions of heat stress, it has been shown that the small heat shock proteins Hsp20, protect cellular proteins from aggregation and membrane lipids from destabilization, in some thermophilic archaea^49^. In taxa of these geothermal sources, these proteins could help the cells to counteract the deleterious effects of environmental stress and in particular of thermal stress. In addition, reverse gyrase coding sequences were found in 29 out of the 42 MAGs; these enzymes are known to be exclusive to hyperthermophiles and involved in DNA protection and repair at high temperatures^50^. Only MAGs RB10-MAG04, RB10-MAG09, RB10-MAG11, RB13-MAG01, RB13-MAG05, RB13-MAG09, RB13-MAG13, RB32-MAG02, RB32-MAG04, RB32-MAG08, RB32-MAG10, RB32-MAG12 and RB108-MAG03, belonging to the phylum *Chloroflexota*, the family *Sulfurifustaceae* or the genus *Caldipriscus* (GTDB taxonomy), do not encode any reverse gyrase gene. These results suggest the presence of numerous thermophilic and hyperthermophilic prokaryotes in these high temperature hot springs. Further cultural and physiological investigations from samples of these Kerguelen hot springs will be necessary to confirm these statements.

In conclusion, this first metagenomic overview of the microbial diversity of Kerguelen hot springs allowed the assembly of 42 MAGs, from four hot springs. Several MAGs correspond to putative new taxa, namely 13 new putative genomic species and 6 new putative genera affiliated to *Bacteria* and *Archaea* according to GTDB. Based on their genetic potential, these taxa appear to be chemolithoautotrophs and chemoheterotrophs and thus probably involved in the carbon, sulfur, hydrogen and nitrogen cycle. Many of these MAGs are likely to be derived from populations of thermophilic/hyperthermophilic bacteria and archaea. As geographically isolated sites, the Kerguelen Islands are reservoirs of diversity and taxa of novel microorganisms that should be interesting to study the evolution of microbial life and speciation processes. It has been difficult to fully assess the microbial metabolic diversity in these geothermal pools due to the inherent limitations of MAG reconstruction and the state of knowledge of microbial pathways that remains limited. However, these geothermal ecosystems could be reservoirs of biological and genomic novelty. The physiological properties and adaptive mechanisms of microorganisms inhabiting these unique environments will deserve to be examined in detail in the future by implementing large-scale metagenomics, metatranscriptomics and cultural analyses.

## Methods

### Sample collection and major elements analysis in water samples

Water samples were collected from four hot springs during the 2016–2017 austral summer TALISKER field campaign (1st of December–11th of February) organized by the French Polar Institute Paul Emile Victor (https://institut-polaire.fr/en/). Water samples and water samples mixed with surficial sediments aliquots were collected. Water samples were collected in 250ML LDPE Nalgene bottles stored at 4 °C until ionic chromatography analysis. Mixed water and sediment samples were collected aseptically in sterile 50 mL Becton- Dickinson and Company-syringes, then stored anaerobically in sterile glass bottles at 4 °C. Field measurements of fluid parameters were performed using a HI9829 (Hanna instruments) multiparameter calibrated and equipped with sensors allowing the acquisition of pH, temperature (°C), alkalinity (mg/L), and electrical conductivity measurements (mS/cm) (Table S1). The major anions and cations were analyzed at LGL-TPE using ion-chromatography (Methrom ECO IC). A mixture of 3.2 mM Na_2_CO_3_ and 1 mM NaHCO_3_ was used as an eluent for analysis of anions and a chemical suppression module (MSM) was used to suppress the conductivity. For cations, 1.7 mM HNO_3_ was used as an eluent. The anions and cations were separated using analytical columns, Metrosep A Supp5 Guard/4.0 and Metrosep C4 250/4.0, respectively (Table S1).

### DNA extraction and sequencing

Hot spring’s samples analyzed here were originally collected to grow thermophilic taxa. They were stored at 4 °C for 2 years before DNA was extracted. For each hot spring sample, three replicates of DNA extraction were conducted individually, and combined as a composition sample, before the sequencing. DNA was extracted with a standard PCI (Phenol:Chloroform:Isoamyl Alcohol (25:24:1)) protocol, as described elsewhere, from 10 g environmental matrix^51^, with the exception that 50 µM linear acrylamide were added to enhance nucleic acids precipitation (Invitrogen). One negative control was included and contained 10 mL of DNA-free sterile water. Elution of total DNA extracts was performed in 30–50 μL EB buffer (10 mM Tris–Cl, pH 8.5). Nucleic acid solution quality was determined using the NanoDrop 8000 (Thermo Scientific, Waltham, MA, USA) spectrophotometer. Double-strand DNA concentration was measured using the kit Quantifluor dsDNA system. DNA samples were sequenced by NovaSeq 6000 (2 × 150 bp, paired-end reads) technology by the Duke Center for Genomic and Computational Biology (GCB) (https://genome.duke.edu/).

### Sequence processing, metagenomic assembly and binning

Metagenome sequences’ quality were controlled by FastQC (v0.11.9—https://github.com/s-andrews/FastQC) and MultiQC (v1.9—https://github.com/ewels/MultiQC). Sequences were then processed with the snakemake of Anvi’o^52–54^ (v7—https://github.com/merenlab/anvio), filtered by integrated minoche script (v2.8—https://github.com/merenlab/illumina-utils). Next, MetaSpades^55^ (v3.14.1—https://github.com/ablab/spades) was used as genome assembler and Concoct^56^ (v1.1.0—https://github.com/BinPro/CONCOCT) as genome binner with anvi_cluster_contigs function (“all against all” mode). Furthermore, MAGs were manually refined with anvi-refine function. Genome mapping was performed with bowtie2^57^ (v2.4.2—https://sourceforge.net/projects/bowtie-bio/files/bowtie2/2.4.2/) and samtools^58^ (v1.7—https://samtools.github.io/). MAGs’ quality was estimated by Anvi’o and furthermore by CheckM^59^ (v1.1.3—https://ecogenomics.github.io/CheckM/), both with default parameters. Total length, number of contigs, N50, and GC% contents were extracted with anvi-summarize function.

### Taxonomic and phylogenetic inference of metagenomic assemblies and MAGs

According to the standards proposed elsewhere^26^, bins were defined as high-quality (HQ) MAGs (> 90% completion, < 5% contamination, presence of the 23S, 16S and 5S rRNA genes and at least 18 tRNAs), nearly high-quality (NHQ) MAGs (> 90% completion, < 5% contamination, other criteria partially covered), medium-quality (MQ) MAGs (≥ 50% completion, < 10% contamination) and low-quality (LQ) MAGs (< 50% completion, < 10% contamination). MAGs often lack 16S rRNA genes due to their conserved and repetitive nature preventing their assembly^60^, so MAGs of near high-quality could be classified as high-quality MAGs by other authors taking this into consideration. The taxonomic affiliation of the MAGs was first investigated by placing the MAGs in a phylogenomic context. The phylogenetic reconstructions were based on 122 archaeal or 120 bacterial single copy conservative marker genes according to the Genome Taxonomy Database (07-RS207 release) and were constructed using de novo workflow implemented in GTDB-Tk (v1.4.1—https://github.com/Ecogenomics/GTDBTkk)^61,62^. Visualization and trees analyses were done using ARB software^63^.

As the taxonomy proposed by GTDB is new and does not correspond exactly to the one recognized by the International Code of Nomenclature of prokaryotes (ICNP), we also analyzed data according to the rules of the Code and its nomenclature, because at the time of writing this article, SeqCode is being implemented^64^. For this purpose, we implemented a combination of genomic indices classically used for the delineation of the different taxonomic ranks, namely: 16S rRNA gene sequence similarity, average nucleotide identity score (ANI) and average amino-acid identity value (AAI). The approach followed and the results are given in supplementary material (Text S1, Table S2, Fig. S2).

### Metabolic profiling

MAGs were processed with KEGG Decoder script (https://github.com/bjtully/BioData/tree/master/KEGGDecoder) from Anvi’o gene calls tables generated with kegg_kofams and then plotted with R packages (ComplexHeatmap, circlize, RColorBrewer, and dplyrt) to get a general annotation with R (v.3.6.3)^65,66^. A more accurate annotation was performed with Prokka (v1.14.6—https://github.com/tseemann/prokka)^67^ and associated outputs were analyzed by using the Pathway Tools software (v.24.5)^68^ with the MetaCyc database (v.24.5)^69^ to explore in details the putative metabolisms encoded in MAGs or public available genome assemblies. Regarding sulfur metabolisms, for dsr genes, the perl script DiSCo (v.1.0.0, https://github.com/Genome-Evolution-and-Ecology-Group-GEEG/DiSCo) was used on the Prokka protein sequences outputs of each MAG to highlight the specific genes^70^.

## Supplementary Information

Supplementary Information.

## Data Availability

The metagenome bins generated and analyzed during the current study are available in the European Nucleotide Archive (ENA) (https://www.ebi.ac.uk/ena/browser/home), under the Project PRJEB46766 (Table 1).
