# Supplementary material for: A metagenomic insight into the microbiomes of geothermal springs in the Subantarctic Kerguelen Islands
Source: Sci Rep. 2022 Dec 23;12:22243. doi: 10.1038/s41598-022-26299-4 (PMC9789041; doi:10.1038/s41598-022-26299-4)
Supplement: Supplementary file 1 — Supplementary Information. [file 41598_2022_26299_MOESM1_ESM.docx]

**A metagenomic insight into the microbiomes of geothermal springs in the subantarctic Kerguelen Islands**

Maxime Allioux^1^, Stéven Yvenou^1^, Alexander Merkel^2^, Marc Cozannet^1^, Johanne Aube^1^, Jolann Pommellec^1^, Marc Le Romancer^3^, Véronique Lavastre^4^, Damien Guillaume^4^ & Karine Alain^1^*

^1^Univ Brest, CNRS, IFREMER, IRP 1211 MicrobSea, Unité Biologie et Ecologie des Ecosystèmes marins Profonds BEEP, IUEM, Rue Dumont d’Urville, F-29280 Plouzané, France

^2^Winogradsky Institute of Microbiology, Research Center of Biotechnology of the Russian Academy of Sciences, Moscow, Russia

^3^UBO, UFR Sciences et Techniques, UR 7462, Laboratoire Géoarchitecture, Territoires, Urbanisation, Biodiversité, Environnement, France

^4^UJM, CNRS, LGL-TPE UMR5276, F-42023, Saint Etienne, France

*Karine.Alain@univ-brest.fr

**Text S1.** Attempt to classify MAGs on the basis of overall genome relatedness indices (OGRI).

In an attempt to go further than the classification made by GTDB-Tk, and as the taxonomy proposed by GTDB is new and does not correspond exactly to the one recognized by the International Code of Nomenclature of prokaryotes (ICNP), we also analyzed the data according to the rules of the Code and its nomenclature. For this purpose, we performed a tentative classification based on the LPSN taxonomy by using a combination of overall genome relatedness indices (OGRI) classically used with isolates for the delineation of the different taxonomic ranks, namely: 16S rRNA gene sequence similarity, average nucleotide identity score (ANI) and average amino-acid identity value (AAI). We considered the following thresholds for the different taxonomic ranks and sequences/indices considered: i/ on the basis of 16S rRNA sequences, <98.7% for a new species, <94.5% for a new genus, <86.5% for a novel order [1]; ii/ on the basis of ANI, <94–96% for a new species [2], <70.85–76.56% for a new genus [3]; on the basis of AAI, 95–100% for a same species, 65–95% for a same genus and 45–65% for a same family [4]. When the taxonomic affiliation could not be resolved by considering the standard deviation value calculated for the indices, the two possible taxonomic affiliations were indicated in Table S2 with a superscript number. When these indices could not be considered due to the lack of cultivated close relatives, we considered GTDB-Tk classification.

For *Bacteria*, taxonomic, phylogenomic and phylogenetic analyses allowed us to place the MAGs in the following clades: three in the *Thermoflexus* genus from three different springs (RB10-MAG04, RB13-MAG05, RB32-MAG02), one in the *Dehalococcoidales* order (RB32-MAG04), one in the *Thermomicrobium* genus (RB32-MAG08), one in the *Chloroflexales* order (RB32-MAG14), one in the *Ktedonobacteraceae* family (RB108-MAG03), four in the *Hydrogenivirga* genus from the four different springs (RB10-MAG07, RB13-MAG10, RB32-MAG07, RB108-MAG02), two in the *Aquificaceae* family (RB10-MAG12, RB32-MAG11), five in the *Thermus* genus from the four geothermal sources (RB10-MAG08, RB10-MAG11, RB13-MAG09, RB32-MAG10, RB108-MAG01), one in the *Meiothermus* genus (RB13-MAG13), two in the ‘*Candidatus* Caldipriscus’ genus (RB10-MAG09, RB32-MAG12), one in the *Acidiferrobacteraceae* family (RB13-MAG01), three in the Armatimonadetes phylum (RB10-MAG03, RB13-MAG04, RB32-MAG03), and one in the ‘*Candidatus* Patescibacteria’ superphylum (RB32-MAG13).

For *Archaea*, assignations of the MAGs were as followed: three in the *Acidilobaceae* family (RB10-MAG01, RB13-MAG02, RB32-MAG01), two in the *Zestosphaera* genus (RB10-MAG02, RB13-MAG06), four in the *Ignisphaera* genus (RB10-MAG05, RB13-MAG08, RB13-MAG11, RB32-MAG05), three in the *Thermofilaceae* family (RB10-MAG06, RB13-MAG03, RB32-MAG09), three in the *Thermoproteales* order (RB10-MAG10, RB13-MAG07, RB32-MAG06), and one could not be classified with LPSN taxonomy (RB13-MAG12).

Out of these 42 MAGs, 23 corresponded to different taxa at the taxonomic rank of species or higher and were distributed respectively, into 1 known species called *Thermus thermophilus*, 11 new genomic species within the genera *Zestosphaera*, *Thermoflexus*, *Ignisphaera* (×2), *Thermofilum*, *Hydrogenivirga*, *Thermus*, *Meiothermus,* ‘*Candidatus* Caldipriscus’ and *Thermomicrobium*, 4 putative new genera belonging to the families *Acidilobaceae*, *Acidiferrobacteraceae*, *Aquificaceae* and *Ktedonobacteraceae,* 5 putative new families within the order *Armatimonadales* and *Thermoproteales* (×2), *Dehalococcoidales* and *Chloroflexales*, and 1 putative new class within the superphylum ‘*Candidatus* Patescibacteria’.

These analyses have made it possible to refine the classification of the taxa present in these sources but must be considered with care because a MAG is a consensus genome of a population, and not equivalent to a genome from an isolated strain, from a clone [5]. In addition, some of the MAGs investigated here are medium or low-quality MAGs.

**Table S1**. Physico-chemical conditions of the RB108, RB13, RB10 and RB32 geothermal springs. bdl, below detection limit. χ, conductivity.

|  | **Field measurements** | | | | | **Laboratory measurements (mg/L)** | | | | | | | | | | |
| --- | --- | --- | --- | --- | --- | --- | --- | --- | --- | --- | --- | --- | --- | --- | --- | --- |
| **Sample** | **T(°C)** | **pH** | **pH (mV)** | **χ (mS/cm)** | **CaCO_3_ (mg/L)** | **F^–^** | **Cl^–^** | **NO_2_^–^** | **Br^–^** | **NO_3_^–^** | **PO_4_^2–^** | **SO_4_^2–^** | **Na^+^** | **K^+^** | **Mg^2+^** | **Ca^2+^** |
| **RB108** | 101.3 | 9.59 | -111.9 | 3.45 | 83 | 21.00 | 1012.00 | *bdl* | 1.37 | 0.16 | *bdl* | 33.10 | 699.2 | 23.76 | *bdl* | 7.868 |
| **RB13** | 93.0 | 7.61 | -10.9 | 0.166 | 29 | 0.43 | 14.32 | *bdl* | 0.04 | 0.16 | 0.10 | 11.83 | 20.90 | 8.78 | 0.51 | 4.699 |
| **RB10** | 78.4 | 8.70 | -67.7 | 0.149 | 25 | 0.58 | 13.52 | 0.06 | 0.04 | *bdl* | *bdl* | 12.35 | 19.96 | 6.82 | 0.11 | 3.342 |
| **RB32** | 97.0 | 5.79 | 95.1 | 0.086 | 3 | bdl | 5.53 | *bdl* | 0.03 | *bdl* | *bdl* | 14.36 | 7.50 | 3.37 | 0.65 | 0.345 |

**Table S2.** Taxonomic diversity classification of the 42 MAGs according to LPSN taxonomy (<https://lpsn.dsmz.de/>) based on average genome relatedness indices. AAI analysis was used to analyze the taxonomic position of some MAGs; for some of them, the calculated standard deviations did not allow for an accurate and unique classification. In these cases, the possible alternative classifications, considering the standard deviations are presented in Table 2 with a superscript number. Legend: ^1^Possibly a new family considering the standard deviation. ^2^Possibly a new genus considering the standard deviation. ^3^Possibly the family *Armatimonadaceae* considering the standard deviation. ^4^Possibly the family *Thermoproteaceae* considering the standard deviation. ^5^Possibly the family *Dehalococcoidaceae* considering the standard deviation. ^6^Possibly the family *Chloroflexaceae* considering the standard deviation. *: GTDB-Tk classification.

| **MAG ID** | **Domain** | **Phylum** | **Class** | **Order** | **Family** | **Genus** | **Species** |
| --- | --- | --- | --- | --- | --- | --- | --- |
| RB10-MAG01 | *Archaea* | *Crenarchaeota* | *Thermoprotei* | *Acidilobales* | *Acidilobaceae***^1^** | ***Not assigned*** |  |
| RB10-MAG02 | *Archaea* | *Crenarchaeota* | *Thermoprotei* | *Desulfurococcales* | *Desulfurococcaceae* | *Zestosphaera***^2^** | ***Not assigned*** |
| RB10-MAG03 | *Bacteria* | *Armatimonadetes* | *Armatimonadia* | *Armatimonadales* | ***Not assigned****^3^* |  |  |
| RB10-MAG04 | *Bacteria* | *Chloroflexi* | *Thermoflexia* | *Thermoflexales* | *Thermoflexaceae* | *Thermoflexus* | ***Not assigned*** |
| RB10-MAG05 | *Archaea* | *Crenarchaeota* | *Thermoprotei* | *Desulfurococcales* | *Desulfurococcaceae* | *Ignisphaera* | ***Not assigned*** |
| RB10-MAG06 | *Archaea* | *Crenarchaeota* | *Thermoprotei* | *Thermoproteales* | *Thermofilaceae* | *Thermofilum* | ***Not assigned*** |
| RB10-MAG07 | *Bacteria* | *Aquificae* | *Aquificae* | *Aquificales* | *Aquificaceae* | *Hydrogenivirga* | *New* |
| RB10-MAG08 | *Bacteria* | *Deinococcus-Thermus* | *Deinococci* | *Thermales* | *Thermaceae* | *Thermus* | *thermophilus* |
| RB10-MAG09 | *Bacteria* | *WOR-3****** | *Hydrothermia****** | *LBFQ01****** | *LBFQ01****** | *Caldipriscus****** | ***Not assigned*** |
| RB10-MAG10 | *Archaea* | *Crenarchaeota* | *Thermoprotei* | *Thermoproteales* | ***Not assigned****^4^* |  |  |
| RB10-MAG11 | *Bacteria* | *Deinococcus-Thermus* | *Deinococci* | *Thermales* | *Thermaceae* | *Thermus* | ***Not assigned*** |
| RB10-MAG12 | *Bacteria* | *Aquificae* | *Aquificae* | *Aquificales* | *Aquificaceae***^1^** | ***Not assigned*** |  |
| RB13-MAG01 | *Bacteria* | *Proteobacteria* | *Gammaproteobacteria* | *Acidiferrobacterales* | *Acidiferrobacteraceae***^1^** | ***Not assigned*** |  |
| RB13-MAG02 | *Archaea* | *Crenarchaeota* | *Thermoprotei* | *Acidilobales* | *Acidilobaceae***^1^** | ***Not assigned*** |  |
| RB13-MAG03 | *Archaea* | *Crenarchaeota* | *Thermoprotei* | *Thermoproteales* | *Thermofilaceae***^1^** | ***Not assigned*** |  |
| RB13-MAG04 | *Bacteria* | *Armatimonadetes* | *Armatimonadia* | *Armatimonadales* | ***Not assigned****^3^* |  |  |
| RB13-MAG05 | *Bacteria* | *Chloroflexi* | *Thermoflexia* | *Thermoflexales* | *Thermoflexaceae* | *Thermoflexus* | ***Not assigned*** |
| RB13-MAG06 | *Archaea* | *Crenarchaeota* | *Thermoprotei* | *Desulfurococcales* | *Desulfurococcaceae* | *Zestosphaera* | ***Not assigned*** |
| RB13-MAG07 | *Archaea* | *Crenarchaeota* | *Thermoprotei* | *Thermoproteales* | ***Not assigned****^4^* |  |  |
| RB13-MAG08 | *Archaea* | *Crenarchaeota* | *Thermoprotei* | *Desulfurococcales* | *Desulfurococcaceae* | *Ignisphaera* | ***Not assigned*** |
| RB13-MAG09 | *Bacteria* | *Deinococcus-Thermus* | *Deinococci* | *Thermales* | *Thermaceae* | *Thermus* | ***Not assigned*** |
| RB13-MAG10 | *Bacteria* | *Aquificae* | *Aquificae* | *Aquificales* | *Aquificaceae* | *Hydrogenivirga* | ***Not assigned*** |
| RB13-MAG11 | *Archaea* | *Crenarchaeota* | *Thermoprotei* | *Desulfurococcales* | *Desulfurococcaceae* | *Ignisphaera* | ***Not assigned*** |
| RB13-MAG12 | *Archaea* | *Aenigmatarchaeota** | *Aenigmatarchaeia** | CG10238-14* | EX4484-224* | JAHLMO01* | ***Not assigned*** |
| RB13-MAG13 | *Bacteria* | *Deinococcus-Thermus* | *Deinococci* | *Thermales* | *Thermaceae* | *Meiothermus***^2^** | ***Not assigned*** |
| RB32-MAG01 | *Archaea* | *Crenarchaeota* | *Thermoprotei* | *Acidilobales* | *Acidilobaceae***^1^** | ***Not assigned*** |  |
| RB32-MAG02 | *Bacteria* | *Chloroflexi* | *Thermoflexia* | *Thermoflexales* | *Thermoflexaceae* | *Thermoflexus* | ***Not assigned*** |
| RB32-MAG03 | *Bacteria* | *Armatimonadetes* | *Armatimonadia* | *Armatimonadales* | ***Not assigned****^3^* |  |  |
| RB32-MAG04 | *Bacteria* | *Chloroflexi* | *Dehalococcoidia* | *Dehalococcoidales* | ***Not assigned****^5^* |  |  |
| RB32-MAG05 | *Archaea* | *Crenarchaeota* | *Thermoprotei* | *Desulfurococcales* | *Desulfurococcaceae* | *Ignisphaera* | ***Not assigned*** |
| RB32-MAG06 | *Archaea* | *Crenarchaeota* | *Thermoprotei* | *Thermoproteales* | ***Not assigned****^4^* |  |  |
| RB32-MAG07 | *Bacteria* | *Aquificae* | *Aquificae* | *Aquificales* | *Aquificaceae* | *Hydrogenivirga* | ***Not assigned*** |
| RB32-MAG08 | *Bacteria* | *Chloroflexi* | *Thermomicrobia* | *Thermomicrobiales* | *Thermomicrobiaceae* | *Thermomicrobium* | ***Not assigned*** |
| RB32-MAG09 | *Archaea* | *Crenarchaeota* | *Thermoprotei* | *Thermoproteales* | *Thermofilaceae***^1^** | ***Not assigned****^3^* |  |
| RB32-MAG10 | *Bacteria* | *Deinococcus-Thermus* | *Deinococci* | *Thermales* | *Thermaceae* | *Thermus* | ***Not assigned*** |
| RB32-MAG11 | *Bacteria* | *Aquificae* | *Aquificae* | *Aquificales* | *Aquificaceae***^1^** | ***Not assigned*** |  |
| RB32-MAG12 | *Bacteria* | *WOR-3****** | *Hydrothermia****** | *LBFQ01****** | *LBFQ01****** | *Caldipriscus****** | ***Not assigned****^*^* |
| RB32-MAG13 | *Bacteria* | Superphylum ‘*Candidatus* Patescibacteria’***** | *Paceibacteria****** | *UBA6257****** | *HR35****** | ***Not assigned*** | ***Not assigned*** |
| RB32-MAG14 | *Bacteria* | *Chloroflexi* | *Chloroflexia* | *Chloroflexales* | ***Not assigned****^6^* |  |  |
| RB108-MAG01 | *Bacteria* | *Deinococcus-Thermus* | *Deinococci* | *Thermales* | *Thermaceae* | *Thermus* | *thermophilus* |
| RB108-MAG02 | *Bacteria* | *Aquificae* | *Aquificae* | *Aquificales* | *Aquificaceae* | *Hydrogenivirga* | ***Not assigned*** |
| RB108-MAG03 | *Bacteria* | *Chloroflexi* | *Ktedonobacteria* | *Ktedonobacterales* | *Ktedonobacteraceae***^1^** | ***Not assigned*** |  |

**Figure S1**: Phylogenomic trees showing the positioning of the 42 MAGs based on GTDB-Tk classification and GTDB database. A. *Aquificota*; B. *Armatimonadota*; C. *Chloroflexota*; D. *Deinococcota*; E. *Proteobacteria*; F. *Patescibacteria*; G. WOR-3; H. *Thermoproteota*; and I. *Aenigmatarchaeota*. Numbers at branch nodes indicate bootstrap values.

**Figure S2**: (**A)** Histogram representing the average nucleotide identity of the 42 MAGs calculated with FastANI (GTDB-Tk) with respect to closest genomes and MAGs from the GTDB database. (B) Tentative prediction of the taxonomic position of MAGs based on overall genome relatedness indices (OGRI), with respect to cultured microorganisms. We considered the following thresholds for the different taxonomic ranks and sequences/indices considered: i/ on the basis of 16S rRNA sequences, <98.7% for a new species, <94.5% for a new genus, <86.5% for a novel order [1]; ii/ on the basis of ANI, <94–96% for a new species [2], <70.85–76.56% for a new genus [3]; on the basis of AAI, 95–100% for a same species, 65–95% for a same genus and 45–65% for a same family [4]. These analyses indicate that these springs are reservoirs of novel taxa. Nevertheless, they must be considered with care because OGRI are generally used for genome comparisons and a MAG is a consensus genome of a population.

**References**

1. Yarza. P.. Yilmaz. P.. Pruesse. E.. Glöckner. F. O.. Ludwig. W.. Schleifer. K.-H.. Whitman. W. B.. Euzéby. J.. Amann. R.. & Rosselló-Móra. R. (2014). Uniting the classification of cultured and uncultured bacteria and archaea using 16S rRNA gene sequences. In Nature Reviews Microbiology (Vol. 12. Issue 9. pp. 635–645). Springer Science and Business Media LLC. <https://doi.org/10.1038/nrmicro3330>

2. Richter. M.. & Rosselló-Móra. R. (2009). Shifting the genomic gold standard for the prokaryotic species definition. In Proceedings of the National Academy of Sciences (Vol. 106. Issue 45. pp. 19126–19131). Proceedings of the National Academy of Sciences. <https://doi.org/10.1073/pnas.0906412106>

3. Barco. R. A.. Garrity. G. M.. Scott. J. J.. Amend. J. P.. Nealson. K. H.. & Emerson. D. (2020). A Genus Definition for Bacteria and Archaea Based on a Standard Genome Relatedness Index. In S. J. Giovannoni (Ed.). mBio (Vol. 11. Issue 1). American Society for Microbiology. <https://doi.org/10.1128/mbio.02475-19>

4. Konstantinidis. K. T.. Rosselló-Móra. R.. & Amann. R. (2017). Uncultivated microbes in need of their own taxonomy. In The ISME Journal (Vol. 11. Issue 11. pp. 2399–2406). Springer Science and Business Media LLC. <https://doi.org/10.1038/ismej.2017.113>

5. Van Rossum. T.. Ferretti. P.. Maistrenko. O. M.. & Bork. P. (2020). Diversity within species: interpreting strains in microbiomes. In Nature Reviews Microbiology (Vol. 18. Issue 9. pp. 491–506). Springer Science and Business Media LLC. <https://doi.org/10.1038/s41579-020-0368-1>
